# Divergent evolution and molecular adaptation in the *Drosophila *odorant-binding protein family: inferences from sequence variation at the *OS-E *and *OS-F *genes

**DOI:** 10.1186/1471-2148-8-323

**Published:** 2008-11-27

**Authors:** Alejandro Sánchez-Gracia, Julio Rozas

**Affiliations:** 1 Departament de Genètica, Facultat de Biologia, Universitat de Barcelona, Avda. Diagonal 645, 08028 Barcelona, Spain; 2 Institut de Biologia Evolutiva (CSIC-UPF), Passeig Marítim de la Barceloneta, 37-49, 08003 Barcelona, Spain

## Abstract

**Background:**

The *Drosophila *Odorant-Binding Protein (*Obp*) genes constitute a multigene family with moderate gene number variation across species. The *OS-E *and *OS-F *genes are the two phylogenetically closest members of this family in the *D. melanogaster *genome. In this species, these genes are arranged in the same genomic cluster and likely arose by tandem gene duplication, the major mechanism proposed for the origin of new members in this olfactory-system family.

**Results:**

We have analyzed the genomic cluster encompassing *OS-E *and *OS-F *genes (*Obp83 *genomic region) to determine the role of the functional divergence and molecular adaptation on the *Obp *family size evolution. We compared nucleotide and amino acid variation across 18 *Drosophila *and 4 mosquito species applying a phylogenetic-based maximum likelihood approach complemented with information of the OBP three-dimensional structure and function. We show that, in spite the *OS-E *and *OS-F *genes are currently subject to similar and strong selective constraints, they likely underwent divergent evolution. Positive selection was likely involved in the functional diversification of new copies in the early stages after the gene duplication event; moreover, it might have shaped nucleotide variation of the *OS-E *gene concomitantly with the loss of functionally related members. Besides, molecular adaptation likely affecting the functional OBP conformational changes was supported by the analysis of the evolution of physicochemical properties of the OS-E protein and the location of the putative positive selected amino acids on the OBP three-dimensional structure.

**Conclusion:**

Our results support that positive selection was likely involved in the functional differentiation of new copies of the OBP multigene family in the early stages after their birth by gene duplication; likewise, it might shape variation of some members of the family concomitantly with the loss of functionally related genes. Thus, the stochastic gene gain/loss process coupled with the impact of natural selection would influence the observed OBP family size.

## Background

The olfactory system of animals allows individuals detecting enormously diverse information from the external environment, being in most species a fundamental feature for their survival and reproduction. Natural selection, therefore, likely plays an important role in the evolution of olfactory-involved genes. Actually, there is compelling evidence for the action of positive selection in the evolution of these genes, both in insects and in vertebrates [e.g. [1-7]. In addition, olfactory-specific gene families might contribute to the host-specificity shifts occurring in the diversification of super-specialist *Drosophila *species [8,9].

The primary step in the olfactory perception is accomplished by the Odorant-Binding Proteins (OBPs). In spite of the similar global function of insect and vertebrate OBPs, these two protein families are evolutionarily unrelated [10]. In insects, OBPs are small globular proteins that bind odorant molecules (including pheromones) at the pores of the chemosensory sensilla, transporting them through the aqueous lymph, and delivering their ligands near the olfactory receptors (OR) [11,12]. In addition, OBPs might play a role in the olfactory coding [13,14], as well as in the stimulus inactivation [15-17]. While some OBPs co-express in the same individual sensilla, some others have strikingly different expression patterns [18]. Currently, the OBP three-dimensional (3D) structures of several insects have been determined [reviewed in [19]; these proteins share similar folds, although with significant structural differences (protein length, position and conformation of *α*-helices, loops and C-terminus), resulting in diverse solvent access properties.

The *Obp *repertory in the genus *Drosophila *constitutes a multigene family composed by a moderately variable number of members (from 40 to 61 genes) [9,18,20]. Results in [9] have shown that the *Obp *genes evolve through a birth-and-death process; the new members originate by tandem gene duplications and gradually diverge in sequence and likely in function. The *OS-E *(*DmelObp83a*) and *OS-F *(*DmelObp83b*) genes are the two closest paralogous *Obp *members of the *D. melanogaster *genome. These genes, located in the 3R chromosome, are separated by ~1 kb intergenic region and show a highly similar gene structure and protein sequence similarity (the mature protein has 70% amino acid identity) [21]. These genes also co-express in the same specific subset of olfactory sensilla (mainly in the sensilla trichoidea) of the *D. melanogaster *antennal segment 3 [22].

DNA polymorphism and divergence analyses at the *OS-E *and *OS-F *genes in the melanogaster [23] and in the old world obscura [Sánchez-Gracia and Rozas, unpublished data) subgroup species of *Drosophila *have shown that these olfactory genes might have evolved non-neutrally. Nevertheless, no firm conclusions regarding the precise evolutionary mechanism could be drawn; therefore, the specific role that natural selection might play in the evolutionary history of this gene duplication, and especially in the origin and maintenance of the duplicated copies, it is still unknown.

Here, we investigate the mechanisms driving the evolution of the genomic cluster encompassing the *OS-E *and *OS-F *genes (the *Obp83 *genomic region) in 18 species of the *Drosophila *genus. We integrate amino acid and nucleotide-based divergence data, with the analysis of the selective constraints and information of the OBP 3D structure and function, to infer the impact of positive and negative selection in the evolutionary history of these genes. We are especially interested in determining the origin and evolutionary fate of these *Obp *genes within the context of a multigene family submitted to a birth-and-death process. We found that functional differentiation, with an active role of positive selection, might contribute to the *Obp *family evolution across *Drosophila *species. We also show that the evolution of the physicochemical-properties of these proteins suggests that the functional divergence might arise through changes affecting the OBP conformational shift mechanisms, modifying the specificity, sensitivity or accessibility of OBPs to the odorants, to the Ors or to other molecules required to the correct odorant perception.

## Results

### The *Obp83 *genomic region in the genus *Drosophila*

**Figure 1:**
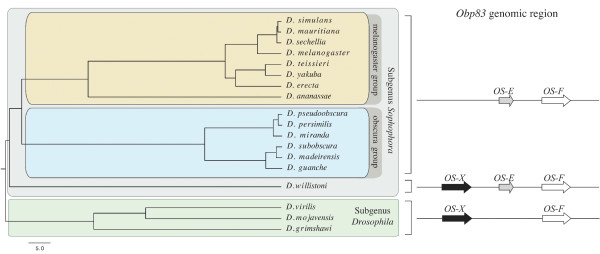
**Structure of the *Obp83 *genomic region across surveyed *Drosophila *species**. The ultrametric tree has been reconstructed using information from [86] and from [87]. Branch lengths are expressed in million of years.

We have identified orthologs of the *OS-E *and *OS-F *genes in the 15 species surveyed of the *Sophophora *subgenus. In all cases, the two genes have the same orientation, well-conserved exon sizes, and show equivalent intron-exon boundary positions and intron phases; furthermore, the close physical distance that these two *OS *genes have in *D. melanogaster *is also conserved across this subgenus. By contrast, the *OS-E *gene is absent in *D. virilis*, *D. mojavensis *and *D. grimshawi*; these species, and also *D. willistoni*, have a new *OS*-like gene, named here as *OS*-*X *gene (*Obp83aL1 *in [9]) (Figure 1). This new gene localizes 5' upstream (~3 kb apart) from the *OS-F *gene, with the same orientation and a very similar gene structure. The dot-plot analyses corroborate that only *D. willisoni *have all these three *Obp *related sequences [see Additional file 1]. These findings, together with the high sequence similarity between the *OS-X *and their neighbour genes (the amino acid identity of the hypothetical OS-X protein with OS-E and OS-F is 53.3% and 61.4%, respectively), and its position in the *Drosophila Obp *phylogenetic tree [9], clearly indicate that *OS-X *is an old *Obp83 *member that arose by a gene duplication event. The exhaustive search across databases allows identifying 5 putative members of the *Obp83 *group [*A. gambiae *OBP17 (XM001231182), *C. pipiens quinquefasciatus *OBP precursor (AAL86413), *C. tarsalis *OBP (AAO73465), and *A. aegypti *OBP1 (AAO74643), OBP3-1 (AAO74645) and OBP3-2 (AAO74646)]. We did not detect any additional *OS*-like members across other available insect genomes.

### Amino acid and nucleotide divergence

**Figure 2:**
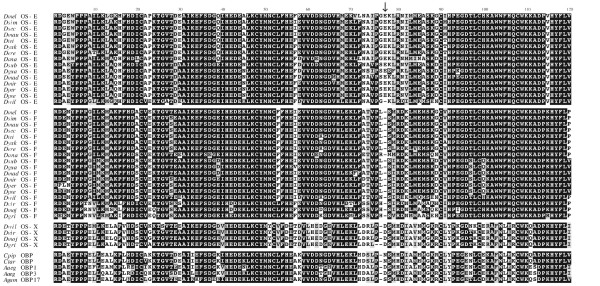
**Multiple sequence alignment of the insect Obp83-like proteins**. Taxa are abbreviated using the first letter from the genus and three of the species name (e.g. *Dmel *indicates *D. melanogaster*). The amino acid positions are numbered relative to the OS-E/OS-F MSA used in maximum likelihood (ML) analyses (i.e., without considering the position with gaps indicated by an arrow).

**Figure 3:**
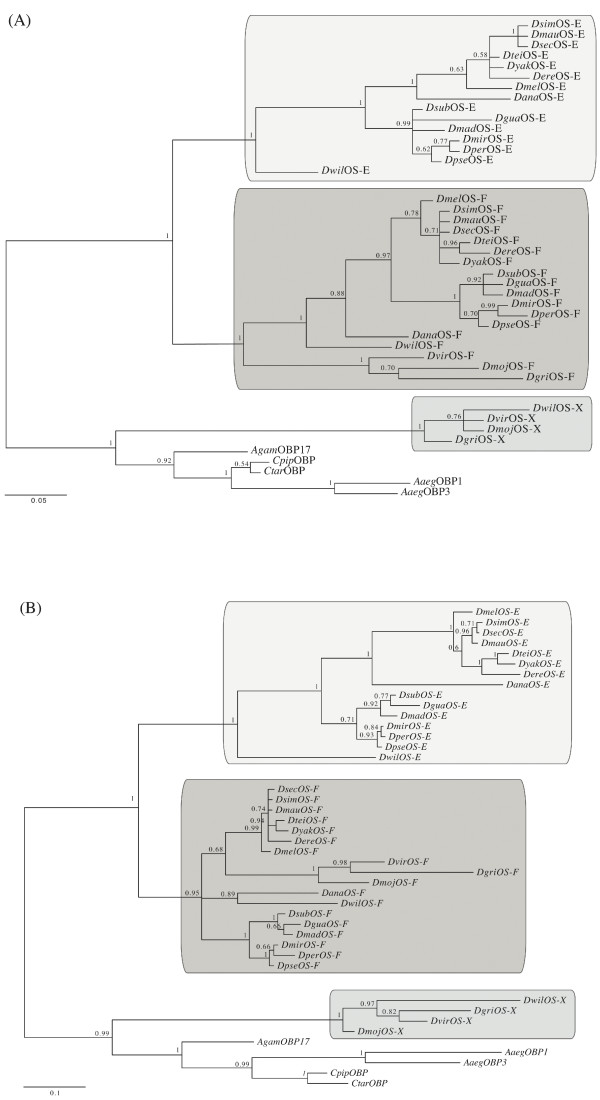
**Bayesian consensus trees of the insect Obp83-like proteins**. Phylogenetic trees based on amino acid (A) and nucleotide (B) sequence data. Each sequence is identified as in Figure 2. The numbers indicate the Bayesian probabilities for each phylogenetic clade. Shaded boxes denote the three *Drosophila *Obp83 orthologs. The scale bars represent amino acid and nucleotide substitutions per site, respectively.

Figure 2 shows the amino acid multiple sequence alignment (MSA) of all OBPs studied. The distinctive six cysteines of the OBP protein family are completely conserved, including those of the *D. erecta *OS-E. Probably, the reported amino acid replacement found in this species [23] would be a specific feature of the sequenced line, as a genetic polymorphism. In overall, the studied OBPs differ in 31.32 amino acids; none of the replacements, nevertheless, would significantly alter the typical secondary structure of the PBP/GOBP protein family (results not shown). Analysis of the synonymous (*K*_S_) and nonsynonymous (*K*_A_) nucleotide sequence divergence across the *Sophophora *subgenus reveals that *OS-E *gene (*K*_A _= 0.049; *K*_S _= 1.040) evolves more rapidly than *OS-F *(*K*_A _= 0.036; *K*_S _= 0.743). Indeed, using the *A. gambiae Obp1 *gene as outgroup, the evolutionary rate differ significantly (RRT; *P *= 0.05). The phylogenetic analysis (Figure 3) shows 3 separated clades, one for each *Drosophila *orthologous group, suggesting an independent evolution since their origin by gene duplication. Estimated gene trees and commonly accepted species tree [[24] and Figure 1] are not completely concordant, likely because of the short sequence length examined; indeed, nodes with discrepancies also have low posterior probability values.

Bayesian trees based on protein and nucleotide divergence indicate that the *OS-E *and *OS-F *genes existed before the split of the *Sophophora *and *Drosophila *subgenera; later, the *Drosophila *subgenera lineage lost the *OS-E *gene (Figure 1). Even using an algorithm more sensitive than BLAST [25], we did not find any vestige of the *OS-E *gene in the genome of the three *Drosophila *subgenus species suggesting, therefore, that this gene has been completely erased. In agreement with previous results [23] the distribution of nucleotide substitutions across the DNA sequence is not homogeneous: both genes are highly divergent in the so called heterogeneous region (region *het 1*, encompassing the amino acid positions 68 to 90 in the *Drosophila *OS-E protein; [23]), and in the first 30 amino acids of the N-terminal part of the mature protein (referred here as *het2*).

### Selective constraints and functional divergence

**Figure 4:**
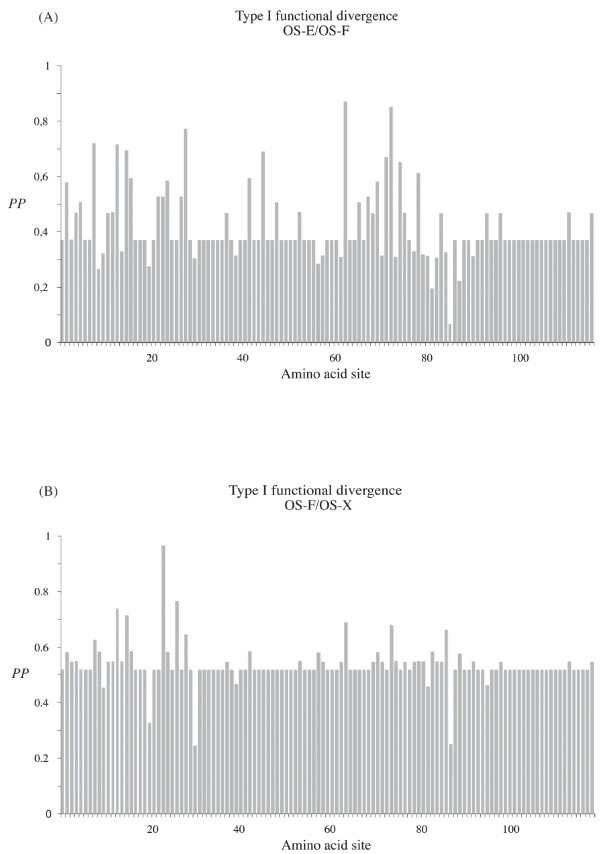
**Type I functional divergence among the *Drosophila *Obp83 members**. Posterior probability (*PP*) profiles of the site-specific type I functional divergence. The positions with gaps involved in each paralogous comparison were not considered.

Gene duplication-specific changes in the substitution rates (type I functional divergence) might reflect variations in selective constraints after gene duplication [26,27]. We detected evidences of type I functional divergence between OS-E and OS-F proteins (*θ*_*λ*I _= 0.411; *P *= 0.006), and between OS-F and OS-X proteins (*θ*_*λ*I _= 0.534; *P *= 0.019); namely, there are some amino acid sites with discrepancies in their evolutionary rate between these paralogous pairs. The OS-E/OS-X comparison was not significant (*θ*_*λ*I _= 0.001; *P *> 0.05). As expected, most amino acids have very low posterior probability (*PP*) values and, therefore, they would not be involved on the hypothetical functional divergence (Figure 4). Specifically, we detected nine and seven amino acid positions (with *PP *threshold values higher than 0.65) in the OS-E/OS-F and in the OS-F/OS-X comparisons, respectively. Besides, five of these functional divergence candidate positions are the same in the two comparisons. We repeated the analysis removing positions with the highest score values, until *θ*_*λ*I _was not significantly different from 0. Using this strongly conservative criterion, the firm candidates to be involved in the functional divergence reduce to two (63 and 73) and one (23) positions, in the previous comparisons. Type I functional divergence may be driven by either site-specific functional constraint relaxations, or by the acquisition of new functions in the one of the duplicated copies. To discriminate between these two hypotheses we infer the functional constraint levels before the gene duplication event by examining the pattern of variation either at the OS-X or the mosquito homologous proteins. We found that unambiguous sites are always more compatible with the relaxation (or loss) of the functional constraint scenario (Figures 2 and 4), and that all three *Drosophila *OBP proteins would exhibit functionally diverged relaxed positions. Interestingly, the putative functional diverged positions shared between the two comparisons always involve relaxations in OS-E or OS-X, but not in OS-F protein. On the other hand, although there are some radical replacements differently fixed between paralogs, we failed to detect any significant type II (cluster-specific) functional divergence.

**Figure 5:**
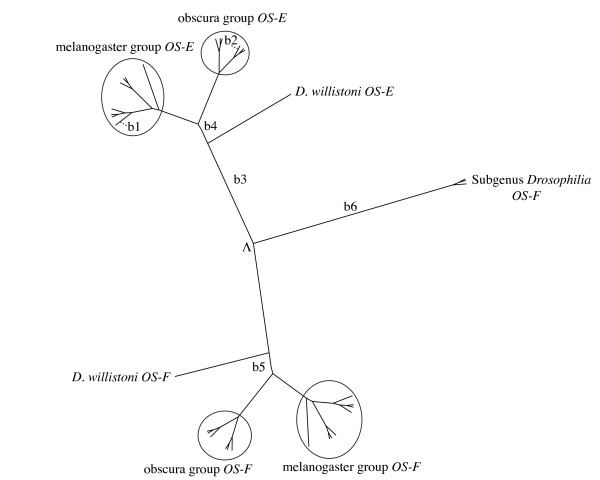
**Unrooted tree with the pre-specified foreground branches used in the ML branch-site analysis**. The *OS-X *sequences were not considered in this analysis (see Methods). b1 and b2 correspond to the *D. melanogaster *and *D. guanche OS-E *external lineages, respectively. Λ, Putative OS-E and OS-F ancestral protein node.

The analysis of the nonsynonymous to synonymous substitution rate ratio can also be used to detect functional differentiation; for this purpose, we used the unrooted tree in Figure 5. Estimates of the overall *ω *ratio from the *OS-E*/*OS-F *MSA (*ω *= 0.064) indicates that purifying selection has been the predominant force acting on the evolution of these *Drosophila *OBPs. Selective constrains, however, are unevenly distributed across the phylogeny (FR model; *P *= 0.020). These differences across lineages, however, are not explained by differences between the duplicated copies (the simplest M0 model fits the data better than the M0dup model, *P *= 0.495). We also detected a significant heterogeneity in the *ω *values across sites (M3 model with three site classes fits the data better than M0 model; *P *= 8.376 × 10^-27^). Nevertheless, the test fails to detect evidences of positive selection across the whole MSA; since positive selection will likely affect a few amino acids at specific lineages on the phylogeny, models estimating *ω *ratios averaged by codons or by lineages are certainly highly conservative. Therefore, we applied an *ad hoc *branch-site approach (using some pre-specified branches, i.e., foreground branches), to assess whether molecular adaptation occurred in the evolution of the *OS-E *and *OS-F *genes in *Drosophila*. Since we had detected, using a polymorphism to divergence comparative analysis, departures from the standard neutral model in the evolution of *OS-E *gene in *D. melanogaster *and in *D. guanche *[[23], Sánchez-Gracia and Rozas, unpublished results], we first evaluated whether the heterogeneity in the selective pressures detected across lineages might be attributed to episodic positive selection events acting on specific positions of these lineages (Figure 5). In spite that null hypothesis of the test 2 (*ω *= 1) was not rejected, the *D. guanche OS-E *gene (b1) exhibits several positions with evidence of relaxed selection (the test 1 was significant) (Table 1); interestingly, many relaxed amino acid positions are located in the highly variable *het1 *region. On the contrary, we did not detect site-specific selective-pressure changes along the *D. melanogaster OS-E *branch (b2). Likewise, we also searched for the specific signature that positive selection associated to the birth-and-death process might leave on coding DNA sequence data. For this purpose, we chose internal branches involving gene gains or losses as the foreground in the branch-site analysis (Figure 5; branches b3, b4, b5 and b6). The analysis shows significant evidences (Table 1) that positive selection might act on specific positions in the early stages after the *OS-E*/*OS-F *gene duplication (after the gene gain; branch b3 in Figure 5), but also on *OS-E *gene concomitantly with the lineage-specific loss of the *OS-X *gene (a member of the same genomic cluster; branch b4 in Figure 5).

**Table 1:** Likelihood Ratio Test (LRT) results and Bayesian prediction of amino acid sites under positive selection.

Foreground^a^	Test-1	Test-2	Positively selected sites^c^
	*P*-value	*P*-value^b^	BEB (*PP *> 0.95)
b1	< 0.001	0.140 (0.070)	74, **75**, **78**, **79**, **81**, **82**, 94
b2	0.084	-	-
b3	< 0.001	0.002 (0.001)	4, **78**, 115, 120
b4	0.002	0.031 (0.015)	**23**, **86**
b5	0.114	-	-
b6	0.994	-	-

Results of the Branch-site analysis.

^a ^Lineages defined as foreground in the branch-site analysis (Figure 5). ^b ^The number in parenthesis indicates the *P*-values under the one-sided test with the asymptotic null distribution (a 50:50 mixture of point mass 0 and *χ*^2^) in [77]. ^c ^Amino acid position numbering according to the MSA in Figure 2. Amino acid positions involving changes in the physicochemical properties influenced by positive selection are highlighted in bold. BEB, Bayes Empirical Bayes results [78].

**Figure 6:**
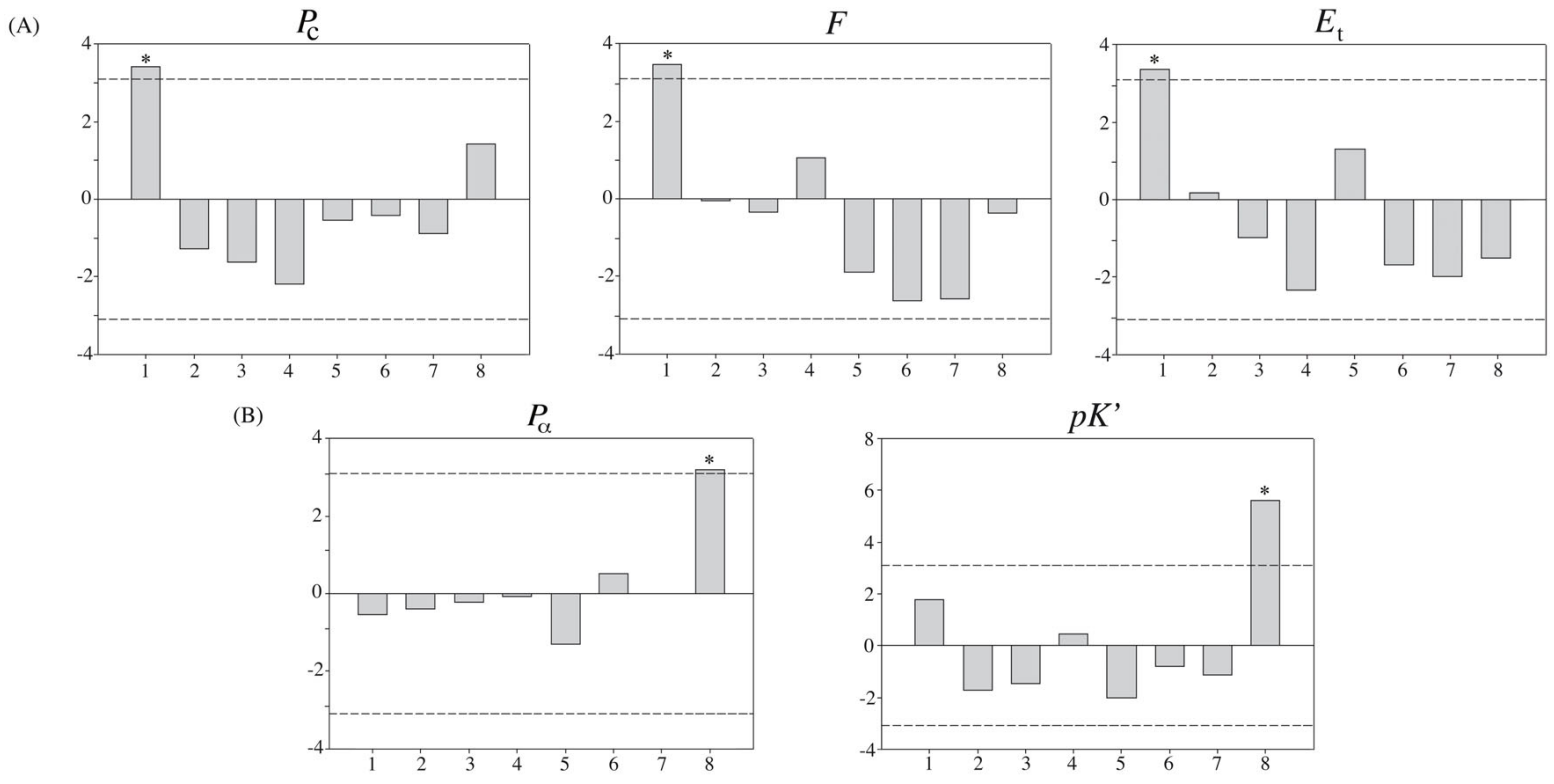
**Amino acid properties affected by positive selection**. Amino acid properties affected by positive-destabilizing (A) and positive-stabilizing (B) selection. *P*_c_, coil tendencies; *F*, mean r.m.s. fluctuation displacement; *E*_t_, total non-bonded energy; *P*_*α*_, *α*-helical tendencies; *pK*', equilibrium constant. The *z-scores *(Y axis) correspond to the proportion of observed amino acid replacements per magnitude category (X axis) [29]. The dashed lines indicate the critical *z-scores *limits. The significant categories are marked with an asterisk.

The molecular adaptation processes occurred after the gene duplication event were also investigated by comparing the magnitude of the physicochemical changes produced by the observed amino acid replacements with those expected at random [28]. The null hypothesis (selective neutrality) was rejected (goodness of fit tests; *P *< 0.001) for six physicochemical properties: coil tendencies (*P*_c_), equilibrium constant (*pK*'), mean r.m.s. (root mean square) fluctuation displacement (*F*), solvent accessible reduction ratio (*R*_a_), total non-bonded energy (*E*_t_) and turn tendencies (*P*_t_), and involves only the OS-E protein. That is, some amino acid replacements altering these physicochemical properties in the OS-E protein accumulated more (or less) often than expected by chance (likely reflecting fitness differences). Moreover, for each physicochemical property, the distribution of the *z-scores *across 8 magnitude classes [29] indicates that, for some properties, there is a significant excess of non-synonymous substitutions at the most extreme magnitude-classes (Figure 6); these OS-E protein properties, therefore, are likely evolving under positive selection. The *OS-F *gene, on the contrary, seems to evolve under strict neutrality, or under strong purifying selection. Interestingly, the position of some OS-E amino acid replacements contributing to the significant excess observed at individual physicochemical magnitude categories match with positions identified in the branch-site analysis (Table 1).

**Figure 7:**
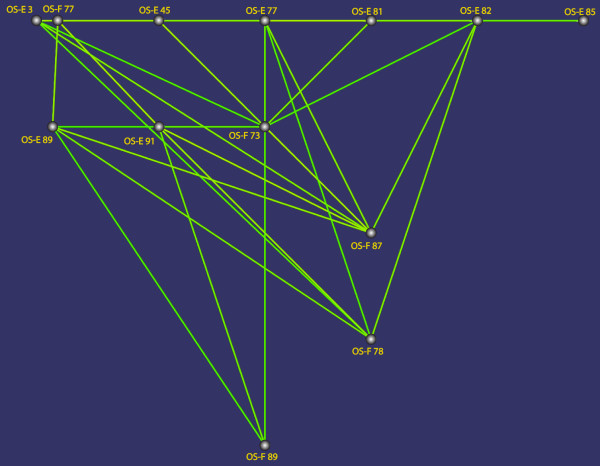
**Representation of the inter-molecular co-evolving amino acids between OS-E and OS-F proteins**. Positions connected by lines correspond to members of the same co-evolving group. The amino acid position is numbering according to the MSA in Figure 2.

Since the *OS-E *and *OS-F *genes are phylogenetically closed and share gene expression patterns [21,22], they might exhibit a coevolving pattern. Indeed, we found statistically significant amino acid covariation between these OBPs (P < 0.001), which might reflect functional or structural constraints. Again, most predicted coevolving positions involve amino acids located in (or close to) the *het1 *region (Figure 7). Outside this variable region, amino acids at positions 3 and 45 of the OS-E protein would be excellent candidates to have co-evolved with amino acids of the OS-F *het1 *region.

### Sequence variation on the OBP 3D structure

**Figure 8:**
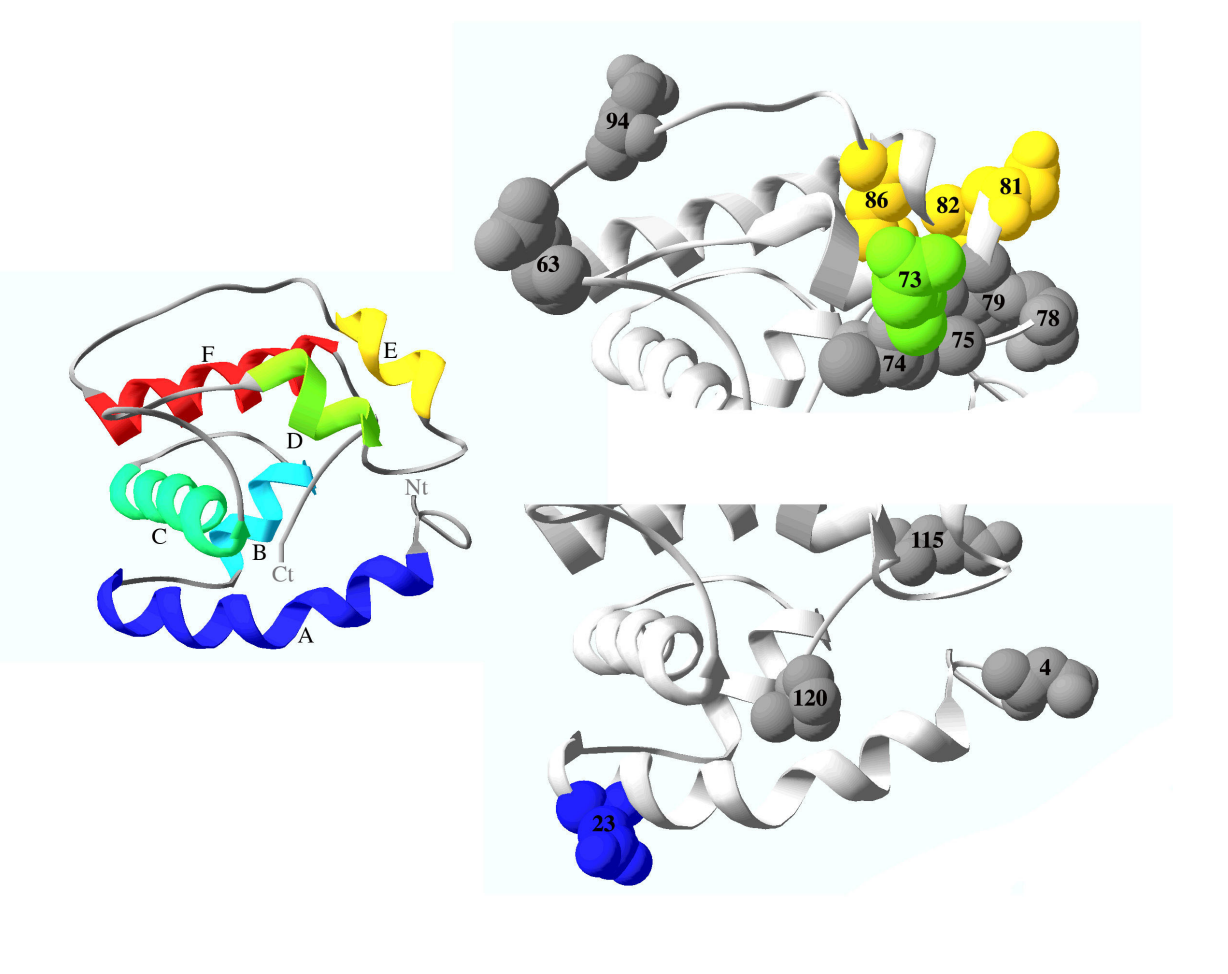
**Location of the amino acid positions identified in the ML analyses on the predicted 3D structure**. Helices, and highlighted amino acids, are depicting with a colour ramp ranging from blue (N-terminal helix) to red (C-terminal helix). The amino acid positions are numbered according to the MSA in Figure 2. Ct, C-terminal end. Nt, N-terminal end.

To determine the functional meaning of the relevant amino acid replacements identified in previous analyses we studied their location in the predicted OBP 3D structure (the putative ancestor of the OS-E and OS-F proteins). We first built an energy-minimized model using a homology modelling approach [30]. The PDB entry with the highest sequence similarity -identified in the PSI-BLAST- corresponds to the *A. gambiae *OBP1 (PDB: 2ERB). We used this entry as a template for the modelling. The *in silico *stereochemical quality analysis [31] indicates that the generated model has a very good quality (the 95.24% of the residues in the most favoured regions), with no residues in disallowed regions. As expected, the modelled structure is roughly similar than the template, with the six helices typical of the OBP family in equivalent positions and with a similar predicted folding (Figure 8).

We first determined the location of the variable regions in the 3D structure; the *het1 *region lies at *α*-helices D and E with their connecting loop, while the *het2 *variable region is at the N-terminal part of the protein, encompassing the complete first *α*-helix. Figure 8 also shows the location in the 3D structure of the relevant positively selected amino acid positions and those inferred to contribute to type I functional divergence (with high *PP *values). Interestingly, most of the selectively relaxed amino acid sites inferred in *D. guanche *OS-E protein and some of the positions responsible of the positive selection are located in the *het1 *region, and therefore nearby in the 3D protein structure. This suggests that this part of the protein has an important functional role. Since the first *α*-helix and the C-terminal end of the protein also appear to be the target of positive selection, molecular adaptation should have affected different OBP protein domains. Otherwise, amino acid positions candidates to be under type I functional divergence are more homogeneously distributed along the 3D structure (results not shown): two of the tree positions with high *PP *values, however, are also in the functionally important predicted protein domains.

## Discussion

### Origin of the *OS-E/OS-F *gene duplication

The *OS-E *and *OS-F *genes encode the two phylogenetic closest odorant binding protein members of the *D. melanogaster *genome [9,20]. We have identified orthologs of the *OS-E *gene only in the *Sophophora *subgenus species, with no evidence in *D. virilis*, *D. mojavensis *and *D. grimshawi *(Figure 1). The analysis in [21] led to the same result in *D. virilis *but they detected two *OS-E *like genes in *Scaptodrosophila lebanonensis*, a basal species of *Drosophila *genus. In fact, in [32] authors proposed that the two *OS-E *and *OS-F *genes have orthologous copies in *A. gambiae*. Our phylogenetically-based analysis support the hypothesis that the *OS-E *and *OS-F *gene duplication predates the *Drosophila *and *Sophophora *subgenera split, but does not agree with [32] findings. Most likely, the *Anopheles *homologous sequences are in fact co-orthologs of the *Drosophila OS-E *and *OS-F *genes, i.e. the *Drosophila *gene copies arose by a gene duplication event after the split of the Nematocera and Brachycera taxa (about 250 Mya). We have to notice, however, that the different evolutionary rates of *OS-E *and *OS-F *genes might constitute a confounding factor. The duplication might have occurred after the *Drosophila*-*Sophophora *subgenus split, followed by evolutionary rate acceleration. This scenario would be consistent with the [21] analysis; these authors, nevertheless, only surveyed a very short fragment (the *het 1 *region), which -in addition- we have found that might have evolved by positive selection. Consequently, the most plausible scenario would indicate that *Obp83 *genomic region had the *OS-X*, *OS-E *and *OS-F *genes before the *Sophophora*-*Drosophila *subgenera split (Figure 1); nevertheless, further experimental analyses using more distant *Drosophila *species would be required for a complete assessment.

### Functional divergence between *OS-E *and *OS-F *genes

Results in [9] have shown that the *Obp *gene family has evolved following a birth-and-death model [33]. Under this model, contrarily to the concerted evolution model, new functional genes often evolve through regulatory or functional differentiation. Accordingly, a number of *Obp *family members differ in gene expression patterns [18,20] or in functional constraints [9]. In the [34] classical view, the functional diversification of duplicated copies is driven by positive selection (i.e. the neofunctionalization model). Otherwise, gene duplicates might also differentiate by acquiring independent sub-functions, being all of them required for carry out the original function. This functional subdivision might be promoted by positive selection [35,36] or be the result of the accumulation of degenerative mutations (i.e., causing a complementary loss of function; the subfunctionalization model) [37]. This later model is, nonetheless, usually considered into the evolution of *cis*-regulatory elements.

In *D. melanogaster*, the *OS-E *and *OS-F *genes have the same temporal gene expression pattern [22,38]. In fact, OS-E, OS-F and LUSH proteins co-localize not only in the sensillar fluid, but also in the same intracellular compartment of the supporting cells before endocytosis [39]. Therefore, it seems unlikely that *OS-E *and *OS-F *genes might differ on the regulatory temporal or spatial pattern of gene expression. Nevertheless, these genes might differ on their quantitative gene expression patterns (unfortunately, the amount of protein produced for each gene is still unknown). It has been shown that gene expression levels are negatively correlated with evolutionary rates [40]. Here we found that the *OS-E *evolves more rapidly than *OS-F*. The highly conserved large first intron (the ~1.7 kb that separate the 5' untranslated and the first coding exon) in all *OS-F *genes might contribute to explain this result. This intron is absent in the *OS-E *genes and has two highly conserved fragments (results not shown), which might contain regulatory regions. This feature, that has been associated with reduced evolutionary rates and high gene expression levels [41], might explain the evolutionary rate differences between *OS-E *and *OS-F *and perhaps putative differences in the gene expression levels.

If the functional diversification between *OS-E *and *OS-F *genes was promoted by changes on the coding region these genes might have evolved with asymmetric evolutionary rates. The *OS-E *and *OS-F *gene duplication is recent enough to allow studying the evolutionary forces acting at the early stages after gene gains in the *Obp *family. Here, we have found that *OS-E *and *OS-F *genes evolved with different substitution rates; nevertheless, the overall functional constraint level (measured as the *ω *parameter) is high and very similar in the two genes. Although relatively ancient duplicates can exhibit similar functional constraint levels [42], they might have differed in a short period of time after the duplication event. In this sense, we have detected both significant type I divergence among OBPs, likely resulting from site-specific relaxations, and the footprint of positive natural selection in the early stages after the *OS-E*/*OS-F *gene duplication. Hence, these two forces would affect the evolutionary fate of new *Obp *family members, initially originated by tandem gene duplications. Since the shifts in the evolutionary rate (detected as type I functional divergence) occurred in different and complementary positions of each duplicated pair, this would point to some functional subdivision, perhaps with a complementary loss (or relaxation) of function at different protein domains. Likewise, we found that positive selection might also act concomitant to some lineage-specific losses of members from the same cluster, suggesting that these within-cluster OBPs should be functionally connected. This functional connection might arise through the formation of OBP dimers at physiological conditions [[43] and references therein]. Natural selection might promote heterodimers via the increase of the combinatory potential of the OBP (increasing either the spectrum of possible target odorants or the binding-specificity) and maintaining, therefore, the co-localization of different OBPs in the same cells. Interestingly, we did not find evidences of functional divergence between OS-E and OS-X proteins, which share a similar functional divergence behaviour with respect to OS-F. Moreover, all extant species (except *D. willistoni*) only have one of these two genes. It is possible that OS-F dimerize with either OS-E or OS-X in the sensillar fluid generating quaternary structures with an equivalent functional role. Although, it is unknown whether in the past OS-E and OS-X really co-expressed in the same cells, it would be very attractive to investigate the expression pattern of the three *Obp *genes of *D. willistoni *(the single species where *OS-E *and *OS-X *currently coexists). The analysis of gene expression data and of the functional quaternary structure might give critical insights into the role of dimerization on the molecular evolution of the *Obp *genes.

### Positively selected sites in the OBP structure

We have detected several sites that likely evolved by positive selection. One of these positions (23), placed in the helix A of the OBP, might alter the size and shape of the binding cavity by modifying the position of the first disulfide bridge [19,44]; for instance, *D. melanogaster *LUSH, which has the first *α*-helix in a more internal position than in *A. mellifera *ASP1, has also a small binding cavity. We also detected putative positive selected sites located in the C-terminal end of the protein (115 and 120). In ASP1 this domain folds inside the protein forming one binding cavity wall and contains residues that interact directly with the ligand; conformational changes in this part of the protein trigger the ligand release close to the odorant receptor [45,46]. Moreover, in LUSH, amino acid substitutions in this part of the protein have also been related with the pheromone-induced conformational shift that triggers the firing of pheromone-sensitive neurons [14]. Interestingly, the structure of this region is the most divergent among the 4 OBPs with resolved 3D proteins: it presents different lengths, secondary structure, or it is even missing. Likely, replacements in these two regions can significantly affect the conformational and the ligand-binding properties of the OBPs, being therefore a major target for adaptive changes.

The third protein region that might be shaped by molecular adaptation comprises the *α*-helices D and E (Figure 8). This region includes both hydrophobic residues covering the binding cavity and exposed amino acids that might be involved in protein-protein interactions. Noticeably, most of the residues (7 out 9) detected in the coevolution analysis (Figure 7) lies in this part of the protein and are exposed to the solvent. Indeed, in the proposed *A. gambiae *OBP17 homodimer, the dimeric interface primarily engage the fourth and fifth helices [47]. Moreover, in this same region also localize many *D. guanche *replacements with a distinctive evolutionary pattern (Table 1). Several authors suggested [48,49] that the very small effective population size of this insular-endemic species would increase the fixation probability of slightly deleterious mutations (as "unpreferent" synonymous mutations, or amino acid replacements). Under this scenario, the amino acid replacements detected in the OS-E protein of *D. guanche *would be slightly deleterious mutations fixed by genetic drift and, therefore, would indicate a functional constraint relaxation rather than positive selection. Moreover, the selective relaxation might be related with a ecologically driven speciation, as has been suggested in other *Drosophila *species [8,9]. Actually, discriminating between positive and relaxed negative selection is not an easy task; even so, since the *het1 *region is functionally important we cannot completely discard that positive selection might in fact also drive the evolution of *D. guanche OS-E *gene.

### Physicochemical evolution and molecular adaptation

The evolutionary analysis of the physicochemical properties might provide insights into the functional divergence occurred across OBPs. We show that OS-E/OS-F duplicates have a markedly different behaviour. While the OS-F protein has been largely affected by purifying selection, both stabilizing and adaptive positive selection was inferred for the OS-E. Indeed, we identified the footprint of the positive-destabilizing selection on two physicochemical properties (Figure 6). *P*_*α *_is a conformational property [50] related to the length and flexibility of alpha helices and, therefore, to the accessibility of specific amino acids (as those involved in interacting motifs of the protein). OBPs are small globular *α*-helical proteins and, therefore, could be largely affected by these changes. *pK*' influences the association and disassociation constants of amino acids, affecting the protein-protein or protein-ligand interactions characteristics. Thus, positive selection might promote functional divergence modifying the binding specificities, or altering the conformational changes involved in the OBP functional mechanism [e.g. [14,45,46,51]]. Additionally, our results also indicate that positive stabilizing selection has acted on the OS-E protein evolution. It has been shown that in globular proteins two (*P*_c _and *F*) of the three physicochemical properties related to positive stabilizing selection are highly-negatively correlated with protein compressibility [52], and would be essential in maintaining the globular structure and the buried nature of the binding-cavity. In spite that the radical changes detected in our study likely produce important functional changes between the OS-E and OS-F proteins, we cannot discard that other detected conservative replacements also have an important adaptive role [see for example [53]]. Indeed, some of the conservative changes contributing to the excess of amino acid replacements detected in the OS-E protein might cause weakly but relevant functional changes in either the OBP binding-activity or in ligand-specificity suggesting, therefore, the action of adaptive instead of stabilizing selection.

## Conclusion

The comparative genomic analysis of the *Obp *multigene family in *Drosophila *[9] has revealed that a birth-and-death model could explain the differences in the number of *Obp *members across species. Here we found that molecular adaptation can also play an important role in the evolution of this olfactory gene family. Indeed, positive selection was likely involved in the functional differentiation of new copies in the early stages after the gene duplication event; likewise, it might shape variation of some members of the family concomitantly with the loss of functionally related genes. The stochastic gene gain/loss process coupled with the impact of natural selection would determine the observed family size [see also [54]]. Nevertheless, further functional experiments would be required to demonstrate the adaptive character of the amino acids inferred as positively selected. The analysis of the non-coding flanking regions is also critical; particularly the putative regulatory sequences of the *OS-E *and *OS-F *genes to investigate putative fine-tuning differences in their gene expression patterns. All these studies and experiments will certainly contribute to better understand the precise role of natural selection and molecular adaptation in the evolution of chemoreception.

## Methods

### Fly samples and databases

We studied 18 species of the *Drosophila *genus (15 and 3 species of the *Sophophora *and *Drosophila *subgenera, respectively; Figure 1). We used highly inbreed lines (10 generations of sib mating) of *D. teissieri*, *D. yakuba*, *D. ananassae *(species kindly provided by F. Lemeunier), *D. pseudoobscura*, *D. persimilis*, *D. miranda *(species kindly provided by R. C. Lewontin), *D. subobscura, D. guanche*, and *D. madeirensis *(species available in our laboratory). In addition, we also analyze DNA sequence data from *D. melanogaster *(AJ574644), *D. simulans *(AJ567753), *D. mauritiana *(AJ563750) and *D. erecta *AJ574775-AJ574776 [23].

### DNA extraction and sequencing

Total genomic DNA of *D. yakuba*, *D. teissieri*, *D. ananassae*, *D. pseudoobscura*, *D. miranda*, *D. persimilis*, *D. guanche*, *D. madeirensis *and *D. subobscura *was extracted from live flies by using a modification of protocol 48 in [55]. DNA fragments, including the complete coding region of the *OS-E *and *OS-F *genes, were amplified by using the PCR protocol [56]. In addition to the primers previously used for the amplification of the *OS*-region in *D. melanogaster*, *D. simulans*, *D. mauritiana *and *D. erecta *[23], we designed additional oligonucleotides for the amplification of the new species. Some of these primers were designed using information of conserved genomic regions between *D. pseudoobscura *and *D. melanogaster *(Berkley *Drosophila *Genome Project, Release 4; [57]). Although the length of the amplified genomic regions varied among species, they always included the coding region of the two genes. PCR products were purified using the QIAquick PCR purification kit (QIAGEN, Chatsworth, CA), and cycle sequenced using primers separated at intervals of ~400 nucleotides. Occasionally, a genome walking strategy was also required to complete the DNA sequence [58]. Sequenced fragments were separated on the ABI 377 and 3700 sequencers. For all species, the DNA sequence corresponding to the coding regions was determined on both strands. The new sequence data have been deposited in the EMBL Nucleotide Sequence Database under accession numbers: FM210093–FM210110.

### Available genomic data sources

We searched for the orthologous (and other homologous) copies of the *OS-E *and *OS-F *genes in other *Drosophila *species using available genome sequence information: *D. sechellia*, *D. willistoni*, *D. virilis*, *D. mojavensis *and *D. grimshawi *[24]; in other insects with sequenced genomes: *Aedes aegypti*, *Anopheles gambiae*, *Apis mellifera*, *Bombyx mori*, *Culex pipiens *and *Tribolium castaneum *[59-63]; http://www.broad.mit.edu/annotation/genome/culex_pipiens/Home.html; and in available sequences from public databases http://www.ncbi.nlm.nih.gov. The orthologous relationship was inferred by the TBLASTN reciprocal best-hit method with further gene trees and species trees reconciliation. Proteins with an amino acid sequence identity higher than 21.5% (the average amino acid sequence identity between OS-E and OS-F and the DmelObp69 protein – the phylogenetically closest OBP in the *D. melanogaster *genome [9]) were proposed as members of the Obp83 subfamily. We determined the gene structure features of newly identified genes using information of the already known *Obp *genes as a guide. To better characterize the syntenic region in the new species, we compared -using the dot-plot method implemented in zPicture tool [64]- the *Obp83 *region (~10 kb) of *D. melanogaster *with the orthologous counterpart in the new species. The SIM local alignment software [24] and the LANLVIEW tool [65] were used to search for possible *OS-E *gene vestiges in the genomic DNA sequence of the *Drosophila *subgenus species.

### Codon sequence analyses

We used SeqMan version 5.53 (DNASTAR, Inc.) for assembling the new sampled DNA sequences. DNA sequences of the coding regions obtained experimentally plus those retrieved from the public databases were multiple aligned using the MUSCLE software [66], and edited with MacClade version 3.05 program [67]. We estimated nucleotide sequence variation by using DnaSP version 4.10 [68], and MEGA version 4 [69] programs. Bayesian phylogenetic analysis was performed with MrBayes version 3.1.2 [70]; for that we applied the best substitution model estimated using the Akaike Informative Criterion implemented in MODELTEST 3.7 [71]. To determine if the paralogous genes evolve at different substitution rates we conducted the two-cluster Relative Rate Test (RRT) implemented in the LINTRE package [72].

We estimated the selective pressures acting on coding regions applying a phylogenetic-based Maximum Likelihood analysis. Since the new homologous *Obp83 *sequences identified in *Drosophila *and in mosquito are highly divergent from the *OS-E *and *OS-F *genes they might differ significantly in nucleotide composition or codon frequencies. This feature might violate some assumptions of the Markov model of codon substitution, and therefore, might yield unreliable estimates of the relevant parameters. To minimize this problem, we used a multiple sequence alignment with information of only the *OS-E *and *OS-F *genes. Given that the alignment of the signal peptide region was unreliable in most homologous gene copies we analyze only the mature-protein coding region. ML estimates of the relevant parameters -as branch lengths and the ratio of the nonsynonymous (*d*_N_) to synonymous substitution rates (*d*_S_), *ω *= *d*_N_/*d*_S_- were obtained using the *codeml *program implemented in the PAML package version 4 [73]. The *ω *parameter was used as a measure of the protein selective constraints [74]. These analyses were conducted under different competing evolutionary hypothesis. We first investigate whether the distribution of selective constraints acting on the *OS-E *and *OS-F *genes fluctuate across lineages; for that, we compared the fit to the data of the "one ratio" model (M0), which assumes a constant selective pressure across branches, with the "free ratios" model (FR), where the rate parameters are estimated independently in each lineage. We also examined other evolutionary scenarios; i) to detect putative changes in the functional constraints after the gene duplication event, we applied a "two clades" model (M0dup) to the data. Under this model we assigned -a priori- two different *ω *ratios, one for each *OS-E *and *OS-F *clades; ii) to assess for site-specific selection pressures (including putative positive selected sites) we used the "site-specific" models (i.e., models that allow variation in the *ω *ratio across sites) of [75]; iii) to detect positively selected sites in specific lineages, we applied the modified branch-site model A of [76] in two consecutive tests (test1 and test2 in [77]); the multiple hypothesis testing problem [78] was taking into account using Bonferroni's correction [79]. The likelihood Ratio Test was used to compare the fit to the data of two nested models, assuming that twice the log likelihood difference between the two models (2Δℓ) follows a *χ*^2 ^distribution with a number of degrees of freedom equal to the difference in the number of free parameters [80]. To prevent incorrect parameter estimates caused by local optima, the *codeml *program was run multiple times for the same model, specifying different initial values.

We used the TreeSAAP version 3.2 [81] to determine the OBP physicochemical properties affected by natural selection. This program compares the distribution of amino acid changes altering a particular physicochemical property (for a set of 31 different properties), with that expected assuming that changes modifying this property are equally likely (i.e., independent of the physicochemical-magnitude change, that is under neutral evolution). For each property the observed and expected distributions are compared by a goodness-of-fit test [28]. TreeSAAP also assign the amino acid substitutions to particular categories in function on their magnitude effect (each property is divided in 8 categories of equal magnitude, from more conservative to more radical physicochemical changes), and determines the statistical deviations from the expected numbers [29].

### Amino acid sequence analyses

Amino acid-based phylogenetic trees were reconstructed using MrBayes. We used the Diverge 2.0 software to estimate the type I (*θ*_*λ*I_) and type II (*θ*_*λ*II_) functional divergence coefficients [26,27] among paralogous proteins. Type I and type II refers to shifts in the substitution rates after gene duplication (indicative of changes in functional constrains), and amino acid replacements completely fixed between duplicates (resulting in cluster-specific alterations of amino acid physiochemical properties), respectively. We used the CAPS program [82] to identify groups of co-evolving positions at intermolecular level; the program also allows estimating the correlated variation between amino acid sites after correcting for evolutionary distances and phylogenetic dependences. The protein secondary structure of the gene products was inferred using the PredictProtein server [83]. We also predicted the putative *Drosophila *OBP 3D structure using the SWISS-MODEL automated modeling server [30]. The putative ancestral sequence of the *Drosophila *OS-E and OS-F proteins (inferred with PAML) was used to search sequences of the Protein Data Bank (PDB) [84] with high amino acid sequence similarity and resolved 3D structure. The 3D structure with the highest PSI-BLAST score was used for the modeling. The Swiss-PdbViewer program version 3.9b2 [85] was used to visualize the 3D structure and to highlight the relevant amino acid replacements identified in the evolutionary analyses.

## Authors' contributions

JR conceived and supervised all research. AS-G obtained and analyzed the data. JR and AS-G wrote the first version of the paper.

## Supplementary Material

Additional file 1 **Dot plots of the *Opb83 *genomic region.** This figure shows the dot plots of the *D. melanogaster Obp83 *genomic region against the orthologous regions of *D. pseudoobscura*, *D. willistoni *and *D. virilis*. Click here for file
