# Supplementary figures and images for: Divergent evolution and molecular adaptation in the Drosophila odorant-binding protein family: inferences from sequence variation at the OS-E and OS-F genes
Source: BMC Evol Biol. 2008 Nov 27;8:323. doi: 10.1186/1471-2148-8-323 (PMC2631505; doi:10.1186/1471-2148-8-323)

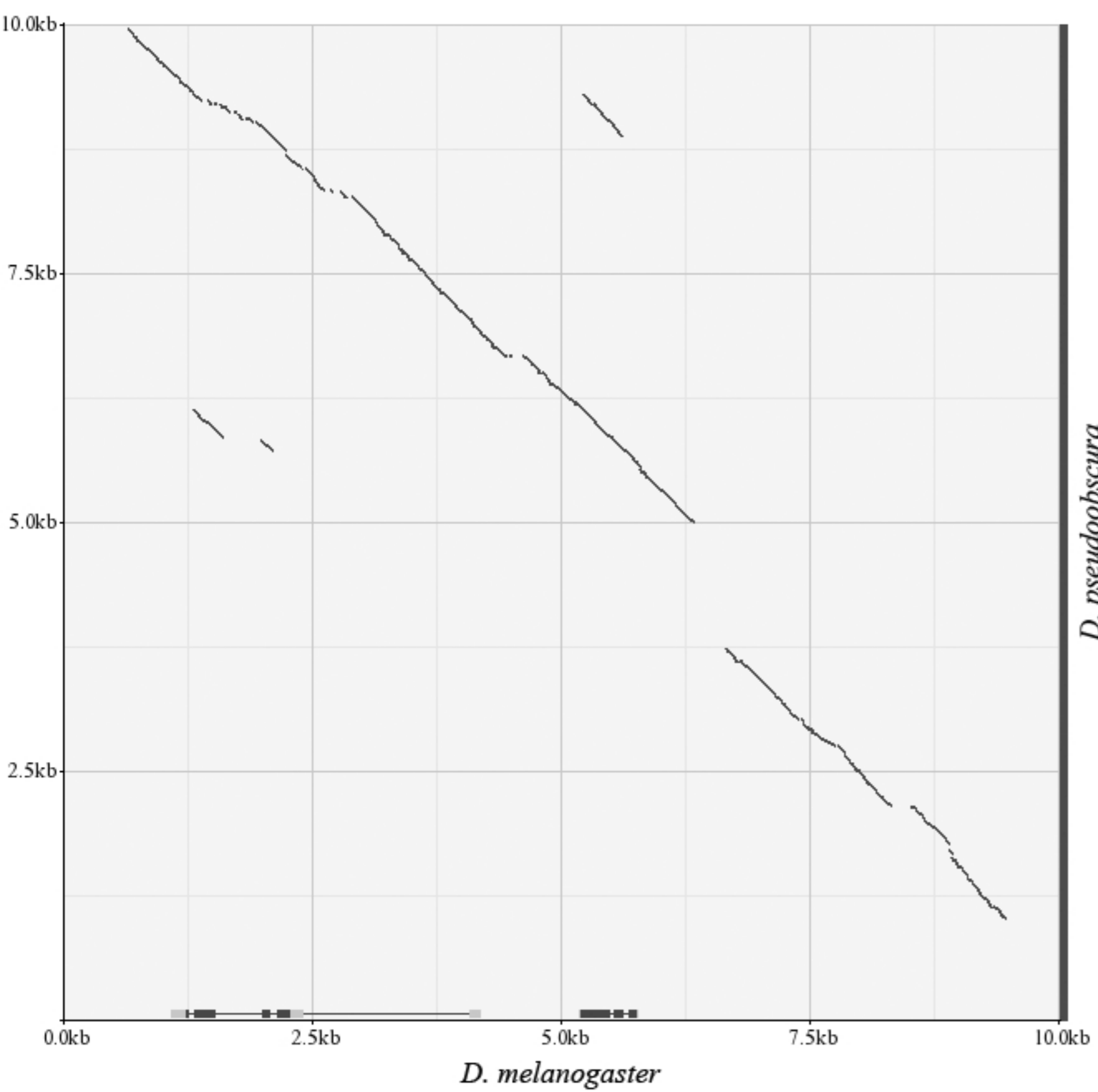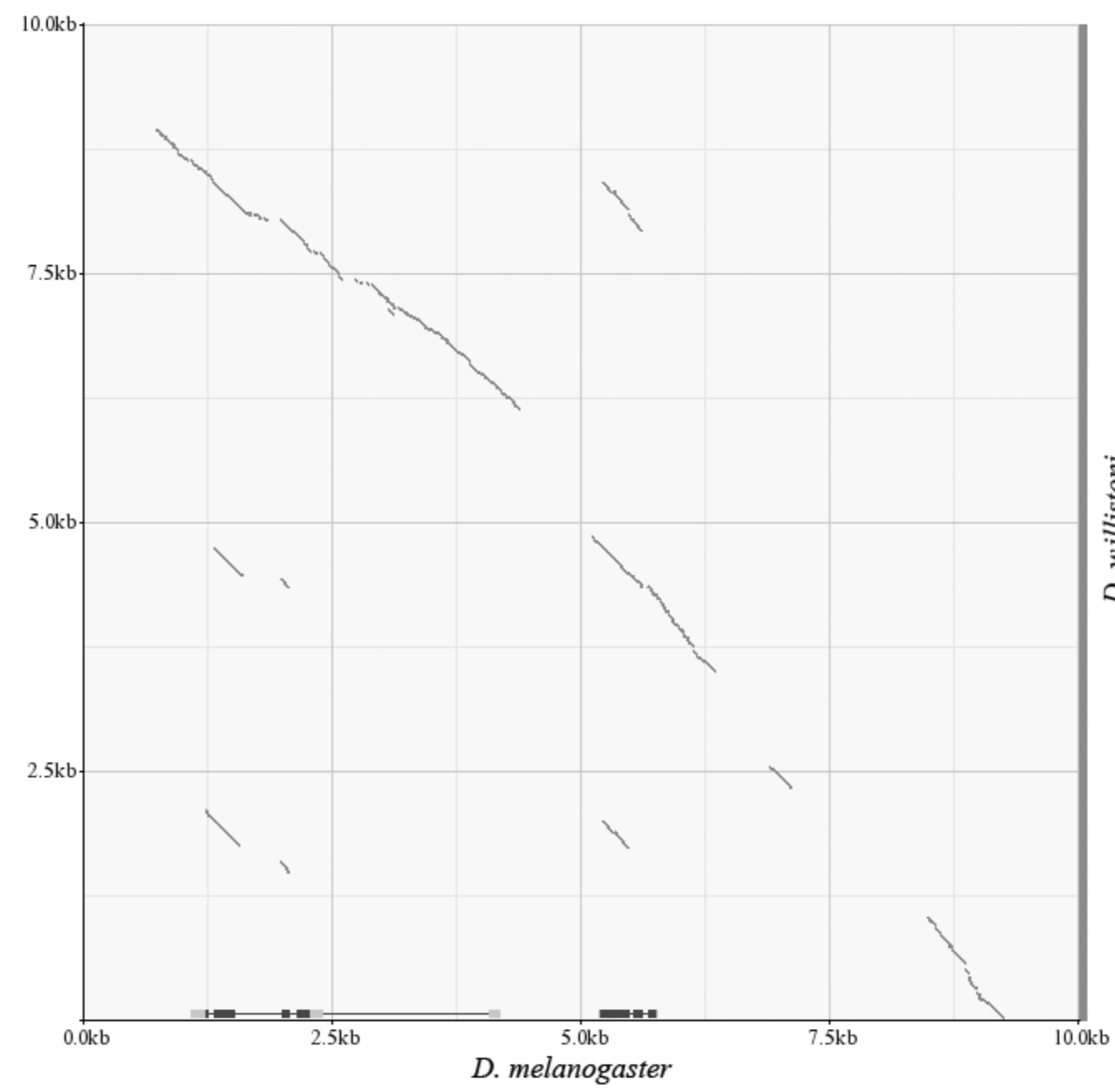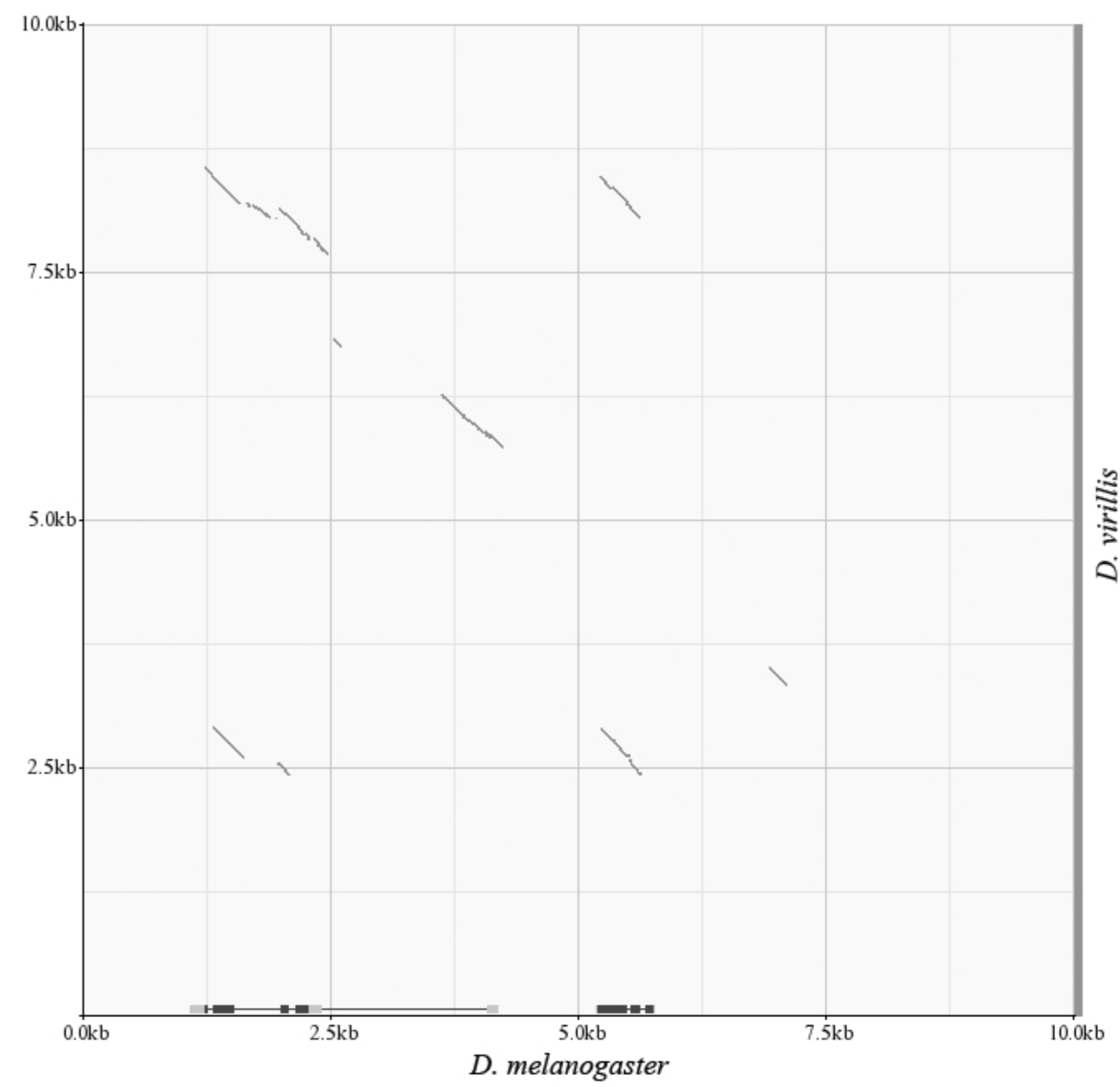

Supplement: Additional file 1 — Dot plots of the Opb83 genomic region. This figure shows the dot plots of the D. melanogaster Obp83 genomic region against the orthologous regions of D. pseudoobscura, D. willistoni and D. virilis. [file 1471-2148-8-323-S1.pdf]
